# The evaluation of bone marrow edema in sacroiliac joint in patients with ankylosing spondylitis using magnetic resonance imaging Dixon sequence

**DOI:** 10.1186/s12891-021-04716-5

**Published:** 2021-11-01

**Authors:** Ming-Shan Du, Xuan-Qi Xiong, He Liu, Xin Qin, Xiao-Fei Hu, Wei Chen

**Affiliations:** 1 Department of Radiology, Southwest Hospital, Third Military Medical University(Army Medical University), Chongqing, 400038 China; 2 Department of Radiology, Chenggu County Hospital, Hanzhong, 723200 China; 3 Department of Radiology, Department of Radiology, Xingyi People’s Hospital of Guizhou Province, Xingyi, 562400 China

**Keywords:** Ankylosing spondylitis, Sacroiliac arthritis, Magnetic resonance, Dixon, Bone marrow edema

## Abstract

**Background:**

Bone marrow edema of the sacroiliac joint is the early imaging manifestation, an indicator of inflammatory activity of ankylosing spondylitis (AS) (Yang R, et. al. Medicine (Baltimore) 98:e14620, 2019).

**Objective:**

The aim of the study was to investigate the value of magnetic resonance imaging (MRI) Dixon sequence in the diagnosis of marrow edema of the sacroiliac joint in patients with AS.

**Methods:**

Forty-five patients with AS admitted in our hospital between November 2016 and February 2019 were selected retrospectively as the case group. Forty-five healthy subjects recruited between November 2016 and February 2019 served as the control group. Bath ankylosing spondylitis disease activity index (BASDAI), Bath ankylosing spondylitis functional index (BASFI), C-reactive protein (CRP) and erythrocyte sedimentation rate (ESR) were recorded after admission to the hospital. The Dixon sequence water-fat ratio of the iliac and sacral surfaces of the bilateral sacroiliac joints in the study group were compared with indicators above in order to find the correlation.

**Results:**

The water-fat ratio under the bilateral sacroiliac joints on Dixon sequence images in the case group was significantly higher than that in the healthy control group (P<0.05). The Dixon sequence water-fat ratio of the iliac and sacral surfaces of the bilateral sacroiliac joints in the study group were positively correlated with spinal arthritis research (SPARCC), BASFI and BASDAI score (all P < 0.05), but did not correlate with ESR and CRP.

**Conclusion:**

The water-fat ratio of magnetic resonance Dixon sequence can be used as a reference index to evaluate the degree of bone marrow edema in active stage of sacroiliac arthritis.

## Background

Ankylosing spondylitis (AS) affects one in two hundred individuals. AS is usually diagnosed many years after onset of symptoms [1]. It is a type of immune-mediated systemic chronic inflammatory disorder, common in adolescents, primarily involving the sacroiliac joint and spine [2]. Therefore, the clinical manifestation is chronic lumbosacral region pain. Severe AS can cause spinal deformity and joint rigidity. Sacroiliac disease or lesions are considered as an earlier indicators of AS. This helps in the early diagnosis and treatment of AS.

The damage of articular cartilage injury and edema of bone marrow under the articular surface is the main pathological manifestation of sacroiliac arthritis in the early stage. It is difficult to find sacroiliac joint lesions using the X-ray in the early period because of the deep sacroiliac joint location, twisted, close joint surface, and large individual differences. However, the introduction of magnetic resonance imaging in sacroiliac joint has improved imaging over the recent years.

X-rays remains the cornerstone of diagnosis of AS. However, MRI has the ability to visualize bone marrow edema and inflammatory lesions within bone in three dimensions. Therefore, diagnosis of AS can be made earlier than when using X-ray to find sacroiliac joint lesions with multi-parameter, multi-sequence, multi-directional imaging and high soft tissue resolution characteristics. With the development of new MRI technology, a growing number of sequences are used in the diagnosis of sacroiliac disease, such as Dixon sequence. Dixon sequence is a quantitative measure of tissue water-fat ratio imaging sequence.

Bone marrow edema (BME) is defined as an area of altered signal on the magnetic resonance imaging (MRI) of the bone. Quantitative assessment of BME using MRI is useful in determining the stage of sacroiliac arthritis. However, there are few reports on the application value of Dixon technique in the evaluation of bone marrow edema in sacroiliac arthritis. The purpose of this study was to explore the value of magnetic resonance imaging Dixon sequence in the quantitative analysis of bone marrow edema in the diagnosis of sacroiliac arthritis in patients with ankylosis spondylitis.

## Methods

### Clinical materials

Our study was retrospective. A total of 45 AS patients admitted in our hospital from November 2016 to February 2019 were enrolled for this study. They included 35 males and 10 females (mean age: 26.37±7.22 years, age range: 14-44 years). Inclusion criteria: Clinical AS patient with the diagnostic criteria of the classification of axial spondylo arthropathy recommended by 2010 Ankylosing Spondylitis International Society (ASAS) criteria [3]; No AS treatment was performed in the past 3 months. Exclusion criteria: Patients with contraindications of MRI examination (e.g. claustrophobic, metal implant patient, etc.); Patients with other bone diseases (such as fracture, bone metastasis, myeloma, tumor etc.); Patients with endocrine disorders (such as hyperparathyroidism, diabetes, kidney disease, etc.); Intemperant.

The 45 healthy persons between November 2016 and February 2019 were taken as the control group. The control group was composed of 33 males and 12 females (mean age: 27.10±6.48 years, age range: 15-42 years). There was no statistically significant difference between the research group and the control group in general information (gender, age) (P>0.05). This retrospective study was approved by the Ethics Committee of Southwest Hospital of Third Military Medical University (Army Medical University) [Approval number: BA2014011]. All subjects signed the informed consent.

### Observation targets

After admission, all the patients with confirmed AS (study group) were measured for the Bath Ankylosing Spondylitis Disease Activity Index (BASDAI) and the Bath Ankylosing Spondylitis Functional Index (BASFI) scores. Laboratory examination with C-reactive protein (CRP) and erythrocyte sedimentation rate (ESR) was also performed.

### MR imaging protocol

Both groups underwent bilateral sacroiliac joint MRI non-enhanced scanning, adopted Siemens Trio 3.0T superconducting magnetic resonance scanner and 6 channels body coil. Horizontal axis and oblique coronal position (parallel to the dorsal line S1 and S3) were scanned from front to back. MRI protocols of sacroiliac joint imaging for sacroiliitis traditionally include precontrast T1-weighted, FST2W or STIR images in axial oblique and coronal oblique planes [4]. Scanning sequence used: Fast spin wave T1 weighted sequence (TSE-T1WI) cross-sectional scan; Rapid spin echo fat suppression T2 weighted sequences (TSE-FS-T2WI) in cross-sectional and oblique coronal scans; Short time reverse sequence (STIR) oblique coronal scan; and multi-echo three-dimensional interpolated water-fat separation fast disturbing phase sequence (Dixon-vibe) oblique coronal scan. The scanning parameters were shown in Table 1.

**Table 1:** MRI scanning parameters of bilateral sacroiliac joint

Sequence	TR/ms	TE/ms	Thickness/ms	Matrix	FOV/mm
Dixon-VIBE cor	9.1	1.23/2.48/3.73/4.98/6.23/7.48	2	256×250	408×357
TSE-T1WI tra	974	20	3	512×410	390×390
TSE-FS-T2WI tra	4500	78	3	512×410	400×319
TSE-FS-T2WI cor	4500	78	3	512×512	450×366
STIR cor	3800	308	5	320×300	390×390

### Dixon sequence water signal value measurement

Dixon sequence images of subjects (study group and control group) were analyzed at the post-processing station of MRI. The bilateral subsurface areas of the sacroiliac joint were delineated on the Dixon sequence water image and lipid image of each client. The water image signal value and corresponding lipid image signal value in the delineated area were measured. The water-fat ratio in the delineated area of the sacroiliac joint surface at each level was also calculated.

The sketch scope of the region of interest (ROI) was: Sacro-sacral foramina beyond to below articular surface; Ilium - within 3 cm of the articular surface. In the ROI mapping process, adjacent bone cortex, osteosclerosis area, blood vessels and artifacts were avoided artificially.

**Fig. 1:**
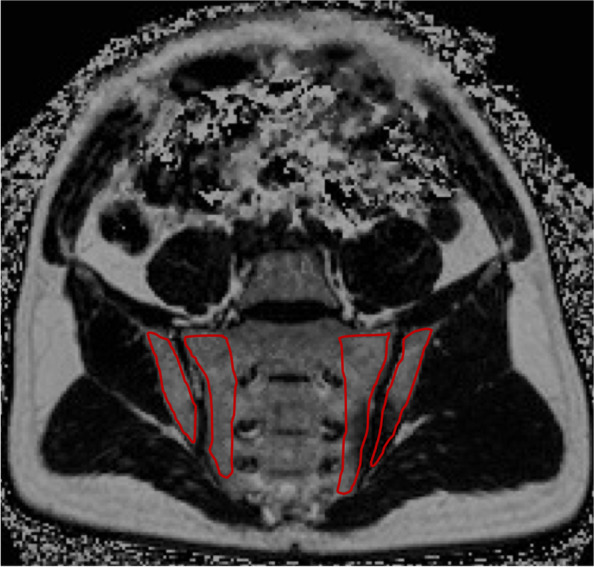
Dixon fat phase ROI sketch diagram. The area drawn in red line is the measurement range

The water-fat ratio was calculated by dividing the water signal value by the sum of the water signal value and the fat signal value, i.e. SW=SIwater / [SIfat+ SIwater] (where SIfat and SIwater are the signal value of water image and fat image, respectively). ROI was measured by repeating the sketch 3 times under the articular surface at each level, and the average value was taken. ROI was plotted and measured independently by two radiologists with similar working years using the double-blind method. The mean value of the measured results was taken as the final result analysis. The consistency of the measured results among observers was evaluated by the correlation coefficient within the group. A typical ROI was outlined in Fig. 1.

### SPARCC score

SPARCC evaluation method: from the oblique coronal position of the sacroiliac joint, 6 continuous planes showing the synovial part are selected for scoring. The sacroiliac joints at each level were divided into 4 quadrants: the upper iliac bone, the upper sacrum, the lower iliac bone, and the lower sacrum. Quadrants showed high signal bone marrow edema focal scores 1 point, without edema focal scores 0 point. When edema focal signal intensity was close to or above the signal intensity of the anterior iliac veins at the same level, the score was increased by 1 point. Additionally, if the depth of the edema lesion extending to the cartilage surface was greater than 1cm, the additional 1 point score was achieved. The maximum of 12 points score was achieved on a single layer, and the total score ranged from 0 to 72 points.

The above two radiologists independently assessed the score of AS patients (the research group) in STIR sequence according to the sacroiliac joint scoring system developed by the Canadian association for spinal arthritis research (SPARCC) [5]. The mean value was used as the final result analysis. The correlation coefficient within the group was used to evaluate the consistency of measurements between observers.

### Statistical analysis

All statistical analyses were performed using SPSS 22.0 statistical software. Intra-class correlation coefficient (ICC) was used to evaluate the consistency of the scores and measurements between the two observers. Homogeneity of variances between groups was determined using homogeneity of variances test. χ^2^ test or Fisher exact test was used to compare the gender differences between the two groups. The independent sample t-test was used to compare the differences in age and water-fat ratio of the sacroiliac joint Dixon sequence between the two groups. Pearson linear correlation was used to analyze the correlation between water-fat ratio of Dixon sequence of sacroiliac joint, clinical score and laboratory inflammatory index, Check level: field = 0.05.

## Results

### General data

There was no significant difference in general information (gender, age) between the two groups (P > 0.05, Table 2)

**Table 2:** Comparison of general data between study group and control group (‾x±s )

Groups	n	Gender (male/female)	Age (years)
Study group	45	35/10	26.37±7.22
Control group	45	32/12	27.10±6.48
P value		0.754	0.972

As measured between the two observers, the intra-group correlation coefficient ICC > 0.900, indicated good consistency. The comparison of Dixon sequence water-fat ratio between the study group and the control group was shown in Table 3.

**Table 3:** Comparison among groups of Dixon water signal values in bilateral sacroiliac joint (‾x±s )

Groups	n	Left sacroiliac joint	Right sacroiliac joint	P value
Study group	45	0.542±0.020	0.544±0.017	0.957
Control group	45	0.438±0.014	0.453±0.014	0.443
P value		0.013	0.011	

**Fig. 2:**
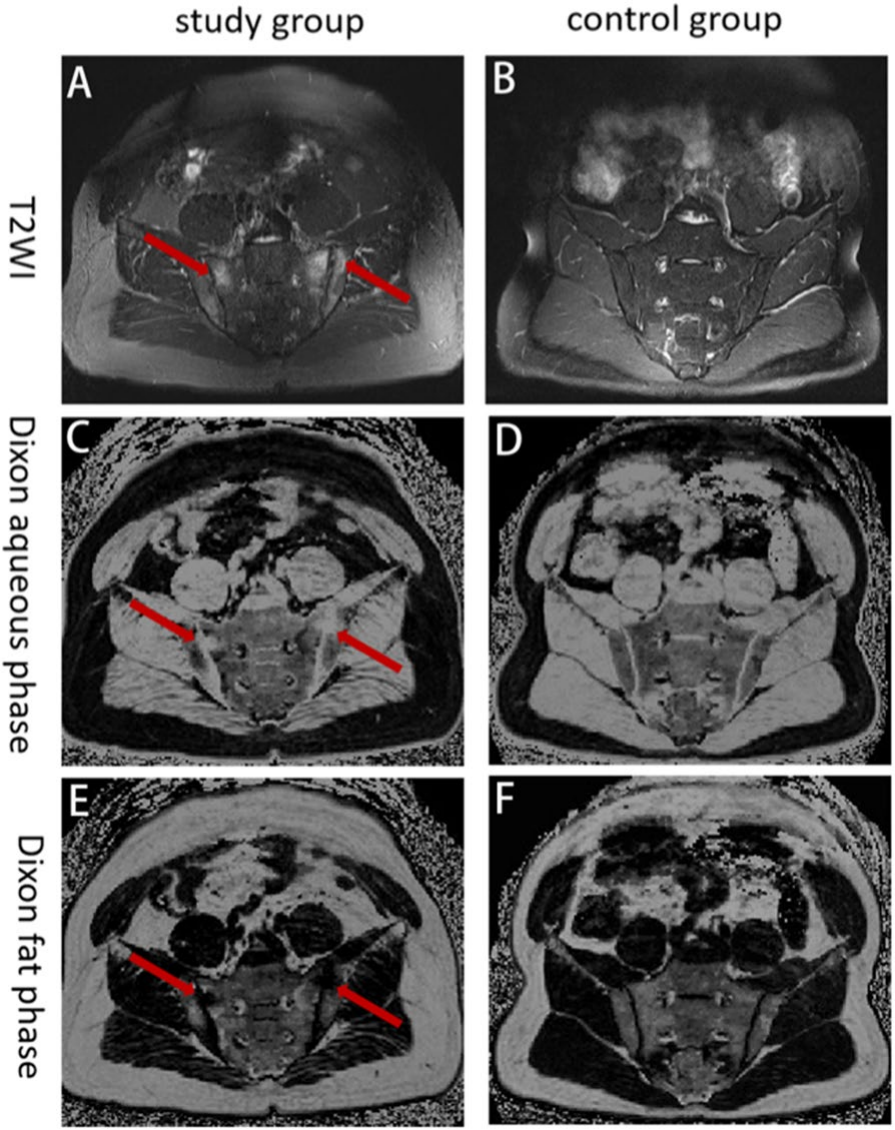
**a** Images of sacroiliac joint T2WI in study group. **b** Images of sacroiliac joint T2WI in control group. **c** Images of sacroiliac joint Dixon aqueous phase in study group. **d** Images of sacroiliac joint Dixon aqueous phase in control group. **e** Images of sacroiliac joint Dixon fat phase in study group. **f** Images of sacroiliac joint Dixon fat phase in control group

The results showed that the Dixon water-fat ratio of the iliac and sacral surfaces of the bilateral sacroiliac joints in the study group was higher than that of the control group. The difference between the groups was statistically significant (P < 0.05). It was suggested that this sequence can be applied to evaluate bone marrow edema in sacroiliac joint. The typical Dixon sequence image of sacroiliac arthritis was shown in Fig. 2.

### Correlation analysis of water-fat ratio of Dixon sequence in sacroiliac joint group

**Fig. 3:**
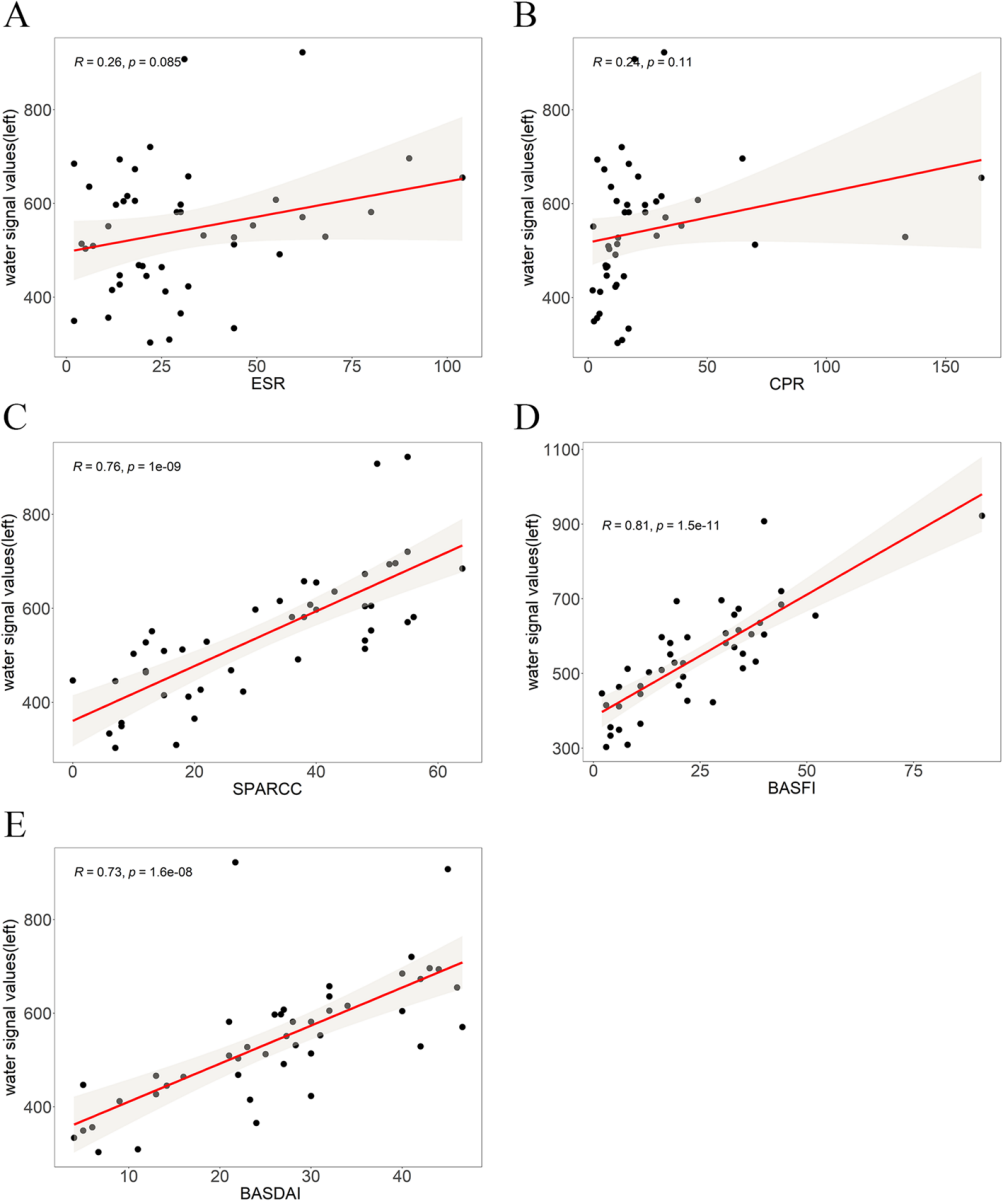
Pearson correlation analysis graph of water-fat ratio(SW) of Dixon sequence in the left sacroiliac joint of the study group with laboratory examination, SPARCC score and clinical score. A to E respectively represent the correlation analysis between Dixon sequence water-fat ratio with "ESR, CRP, SPARCC score, BASFI score, BASDAI score"

**Fig. 4:**
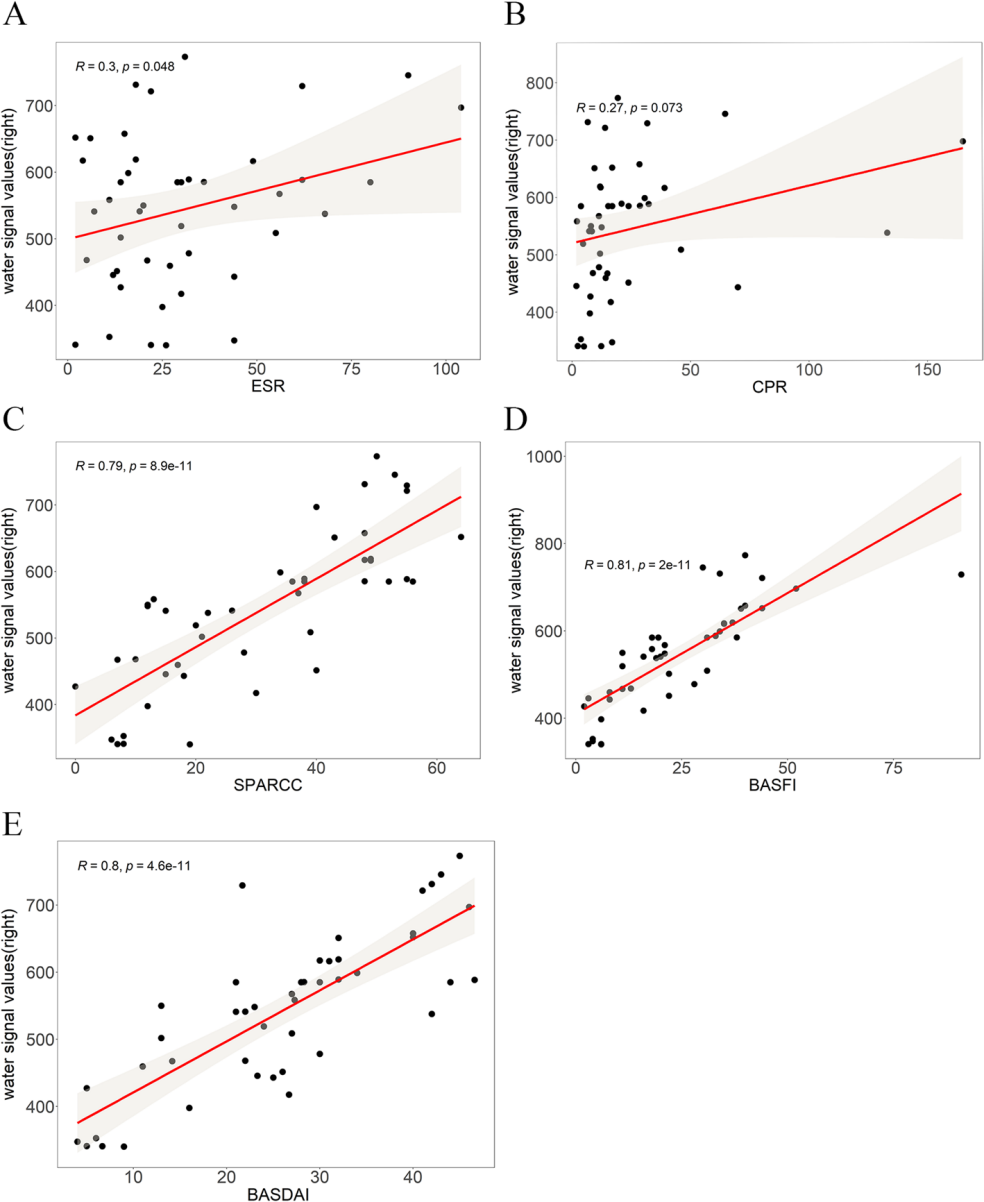
Pearson correlation analysis graph of water-fat ratio(SW) of Dixon sequence in the right sacroiliac joint of the study group with laboratory examination, SPARCC score and clinical score. A to E respectively represent the correlation analysis between Dixon sequence water-fat ratio with "ESR, CRP, SPARCC score, BASFI score, BASDAI score"

The intra-group correlation coefficient (ICC) of SPARCC score between two observers was above 0.9, indicating good consistency. The results of correlation analysis between Dixon sequence water-fat ratio and blood sedimentation, C-reactive protein, SPARCC score, BASFI score and ASDAI score of bilateral sacroiliac joint in the study group were shown in Figs. 3 and 4. The results showed that the Dixon sequence water-fat ratio of the iliac and sacral surfaces of the bilateral sacroiliac joints in the study group were positively correlated with SPARCC, BASFI and BASDAI score (all P < 0.05), but did not correlate with ESR and CRP.

## Discussion

The bone marrow water-fat ratio in the region of interest delineated under the sacroiliac joint surface by MRI Dixon-vibe sequence was measured. The results showed that the water-fat ratio in the ankylosing spondylitis (AS) group were significantly higher than that in the control group (P < 0.05). This suggested that this sequence can be applied to evaluate bone marrow edema in the sacroiliac joint. This will help to diagnose the active phase of inflammation. Therefore, similar to previous studies by koo [6], obvious bone marrow edema exists in early AS, which is displayed using MRI Dixon. During the active phase of inflammation, fat content bone marrow edema is lower than the normal marrow region. As post-inflammatory phase, the proportion of fat stored in the bone marrow will increased accumulation [6]

AS is a chronic inflammatory disease affecting mainly young adults. AS belongs to the group of multiple seronegative spondyloarthropathy. Early and accurate detection of AS is essential for effective disease treatment [7]. Results show that bone marrow edema of the sacroiliac joint is the early imaging manifestation and the symbol of inflammatory activity of AS [8]. Therefore, the early and quantitative detection of bone marrow edema in the sacroiliac joint is crucial for the timely treatment of AS patients, as well as the evaluation of efficacy and the monitoring of pathological activity during treatment.

Currently, BASDAI, BASFI score, CPR and erythrocyte sedimentation rate are mostly used to evaluate the disease activity and severity in clinical practice [9]. However, previous studies have showed that the sensitivity of ESR and CRP to the evaluation of AS was not well [10]. The indicators above have certain limitations. For example, some patients still have pain and other uncomfortable clinical symptoms even with normal ESR and CPR. This shows that ESR and CRP do not objectively reflect the disease state at that time.

Other indicators have their own limitations except for ESR and CRP poor sensitivity. BASDAI and BASFI scores are based on subjective evaluation of condition of the patients, which is affected by many factors. Meanwhile, they are not supported by some quantitative clinical indicators. Some patients have no peripheral joint performance. Therefore, their final score result cannot objectively and accurately evaluate the disease.

The Spondyloarthritis Research Consortium of Canada (SPARCC) and Sacroiliac Joint (SIJ) Structural Score (SSS) methodologies are the most popular imaging standards of active and chronic disease activity, with high accuracy and reproducibility [11]. This SPARCC score is an internationally recognized quantitative indicator used to evaluate the degree of sacroiliac joint inflammatory edema. SPARCC score is based on the semi-quantitative score obtained from the subjective visual examination of the doctor reading the film. It does not provide accurate and objective quantitative signal value of bone marrow edema.

The Assessment in Spondylo Arthritis International Society (ASAS) introduced new criteria for the classification of axial spondyloarthropathies in 2009. MRI has become the major imaging modality for detecting sacroiliitis in the sacroiliac joints [12]. Additionally, phase maps in MRI can reflect local field variation and play a key role in many clinical applications, such as water-fat separation, susceptibility-weighted imaging, and quantitative susceptibility mapping [13].

MRI Dixon-vibe sequence is a quantitative technique based on the separation of water and fat. It makes use of the proton precession frequency difference between water and fat molecule [14]. It adjusts the echo time to do multiple collection, a breath-holding collection of 6 echoes in order to separately calculate the signal values of the water phase and the fat phase [15]. The bone marrow of adults contains about 50% fat and 50% water. When the AS patients are in the active stage of bone marrow edema, the proportion of bone marrow fat content decreases, resulting in the change of the water-fat ratio of Dixon sequence. Bone marrow edema presents with high signal on the water phase for this Dixon-vibe sequence, but low signal on the fat phase. Therefore, the content of bone marrow edema in the delineated area can be quantitatively evaluated more accurately. A quantitative and visible observation index can be provided to clinicians. The proportion of fat deposition was higher as the disease progressed [6]. This was higher than in the normal bone marrow and can be detected using Dixon sequence. In this study, the differences in water-fat ratio between study group and control group were observed. The correlations between the sacroiliac joint surface water-fat ratio in the study group, and BASDAI score, BASFI score, CPR, ESR , and SPARCC score were also analyzed. The water-fat ratio in the study group was positively correlated with BASDAI score, BASFI score, and SPARCC score (all P < 0.05). This suggested that this sequence not only diagnoses the active phase of sacroiliac arthritis, but also can be used as a reference index to evaluate the clinical inflammatory activities. This enables clinicians to obtain more accurate numerical evaluation from the previous semi-quantitative evaluation for patients with inflammatory edema. This provides a more reliable and objective basis for disease staging.

The bony changes are best identified using conventional radiographs [16]. Spinal inflammation is best assessed using MRI. MRI plays an important role in diagnosing, classifying and monitoring the progress of AS inflammation with the ability to provide water-only, in-phase, opposed-phase, and fat-only images in one acquisition [17]. Meanwhile, with the development of new MRI technology, more and more sequences are used to evaluate the therapeutic effect of the sacroiliitis.

Limitations: first, the sample size was small; second, only patients with earlier disease stage were selected in the experimental group. In the later stage, the sample size and the number of patients with late disease stage, should be increased. Intergroup comparison of Dixon sequence can also be performed in patients with advanced stable stage and advanced active stage.

## Conclusion

MRI Dixon-vibe sequence is a relatively accurate quantitative imaging technology. It can be used to identify the disease as early as possible, accurately assess the disease and early block the course of disease development to guide the management and treatment of sacroiliac arthritis. It is expected to become a simple, accurate and effective efficacy observation index which will guide clinical treatment, evaluate efficacy and monitor disease activities. This promotes clinical application.

## Data Availability

The data sets supporting the results of this article are included within the article and its additional files. The datasets are available from the corresponding author on reasonable request.

## Abbreviations

MRI: Magnetic resonance imaging
AS: Ankylosing spondylitis
BASDAI: Bath ankylosing spondylitis disease activity index
BASFI: Bath ankylosing spondylitis functional index
CRP: C-reactive protein
ESR: Erythrocyte sedimentation rate
